# *In vitro* Comparison of Cone Beam Computed Tomography and Ultrasonography Imaging Methods in the Evaluation of Artificial Mandible Intraosseous Lesions

**DOI:** 10.30476/dentjods.2021.87481.1264

**Published:** 2021-09

**Authors:** Numan Dedeoğlu, Şuayip Burak Duman, Oğuzhan Altun, Büşra Arıkan

**Affiliations:** 1 Dept. of Oral and Maxillofacial Radiology, Faculty of Dentistry, Inonu University, Malatya, Turkey

**Keywords:** Intraosseous lesion, Cone beam computed tomography, Ultrasonography, Artificial mandible

## Abstract

**Statement of the Problem:**

Intraosseous lesions of jaws can be imaged by cone beam computed tomography (CBCT) and ultrasonography (USG). The knowledge of imaging features of these two methods about intraosseous jaw lesions is important for dental radiology.

**Purpose:**

The aim of this study is to evaluate artificial mandible intraosseous lesions by using CBCT and USG.

**Materials and Method:**

In this *in vitro* study, intraosseous lesions containing water, milk, olive oil, and liver were evaluated in 60 artificial mandibles by using CBCT and USG. Lesion sizes were compared between CBCT and USG. Lesion sizes were measured on the anterior-posterior, bucco-lingual, and superior-inferior sides. Hounsfield unit (HU) values of the lesions in CBCT images were compared between different materials. Echogenicity of the lesions were evaluated in USG images. One sample t and one-way Anova tests were used for the statistical analysis of the study (*p*< 0.05).

**Results:**

In all size measurements of the lesions, mean CBCT values were statistically higher when compared with USG. In CBCT images, statistically difference was found between the HU values of lesions containing olive oil and other lesion contents. In USG images, echogenicity of water, milk and olive oil was found to be anechoic and the echogenicity of liver was found to be hypoechoic.

**Conclusion:**

CBCT was found to be more accurate than USG in measurement of the size of mandibular intraosseous lesions. According to the results of our study, it was thought that only oil content could be differentiated by using CBCT HU values. It was found that lesions with liquid and non-liquid contents could be differentiated with their echogenicity difference in USG images.

## Introduction

In the radiographic evaluation of jaw lesions, extraoral techniques, panoramic radiograph, periapical, and occlusal techniques from intraoral radiographs can be considered as the first choice [ 1 - 3 ]. Although these two-dimensional methods provide the opportunity to evaluate the maxillofacial bone structure easily and with less radiation dose, they have problems such as the inability to measure the size of the lesions and their relationship with significant anatomic structures accurately in addition to disadvantages such as poor resolution, distortion, and magnification [ 1 , 3 - 4 ]. By employing computed tomography (CT) technique, images with three dimensions, in different planes, without superposition and real dimensions can be obtained from the lesion. Due to these characteristics, CT is considered as the gold standard in the diagnosis and treatment planning of intraosseous lesions [ 1 , 5 - 7 ]. Cone beam computed tomography (CBCT) can be used as an alternative to CT in dental practice and provides very valuable additional information to images obtained with classical methods in the diagnosis, treatment planning, and patients follow-up. It provides three dimensional and high-resolution images of hard tissues with low radiation dose [ 8 - 9 ].

Ultrasonography (USG) has recently been used frequently in maxillofacial imaging and its usage in diagnosis of head-neck lesions has been broadly accepted [ 10 - 14 ]. Literature has shown USG to be a beneficial imaging method in the diagnosis and detection of lesions in jaw bones [ 5 , 15 - 17 ].

The aim of this study was to compare and evaluate CBCT, which is frequently used today in dental radiology to examine radiological features of perforated lesions formed in artificial mandible, with USG, which is used in soft tissue evaluations. 

## Material and Method

**Figure 1:**
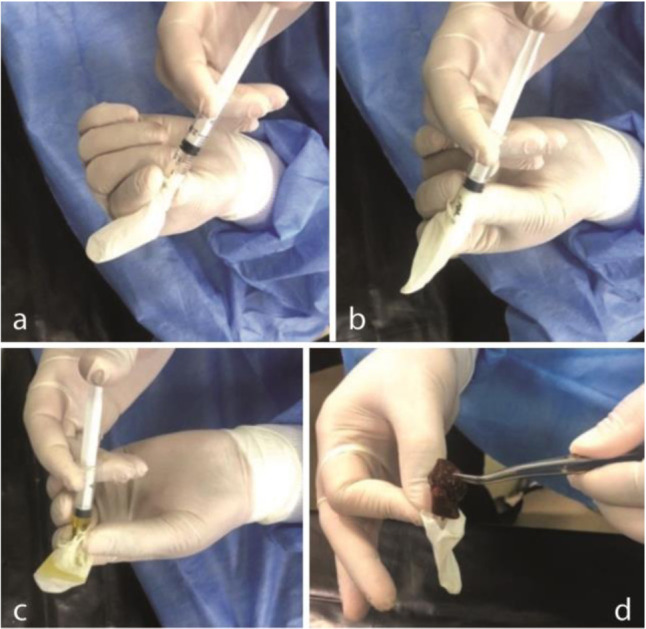
Placing different materials in the bubbles obtained from the finger part of latex gloves to obtain artificial lesion (a: Water, b: Milk, c: Olive oil, d: Liver)

**Figure 2:**
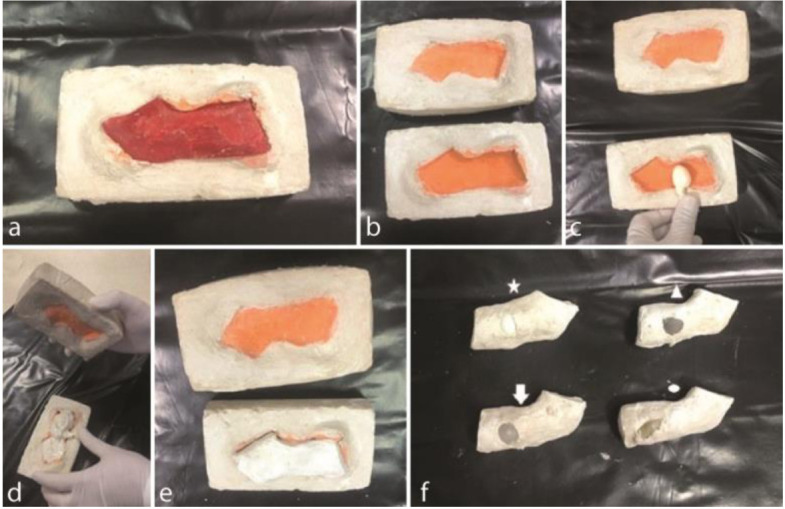
Placing bubbles of artificial lesion in mandible shaped mould and obtaining artificial mandibles including intraosseous artificial lesion, (a and b: Artificial mandible mould, c: Placing the artificial intraosseous lesion in the mould, d: Filling cast in artificial mandible with artificial lesion, e: Taking artificial mandible and lesion out of the mould, f: Perforated lesions including: Asterisk: milk, arrow: water, triangle: liver, circle: olive oil)

For the study, radiological evaluations of 60 artificial mandibles obtained from white plaster cast and artificial intraosseous lesions were made by using CBCT and USG. For artificial lesion, finger parts of latex examination gloves were cut, materials with various intensities were placed inside and bubbles were obtained by tightly tying their openings (Figure 1). For artificial lesion, milk was used in 16 artificial mandibles, water was used in 15, beef liver in 16 and olive oil was used in 13 artificial mandibles. Next, these bubbles were placed in moulds in the shape of a mandible and plaster cast was put into them. After hardening, the perforation area was verified (Figure 2).

### CBCT scanning and analysis of artificial mandible intraosseous lesions

#### CBCT Imaging Procedure

The images of artificial mandible intraosseous lesions used in the study were obtained by using NewTom 5G (Verona, Italy) CBCT machine, with 18x16 field of view, scanning time of 18 seconds, and exposure times of 3, 6 seconds. Evaluation was made with new NewTom software program. In order to determine the lesions border for measurement, the studies were conducted in the dark room.

### Evaluation of CBCT Images

#### Sizes

**Figure 3:**
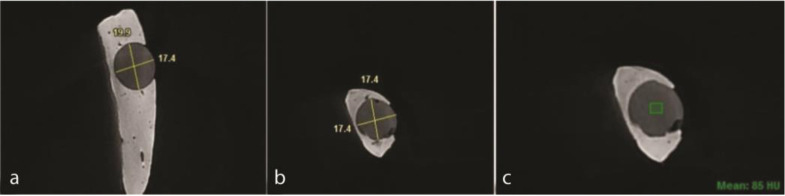
Measurement of lesion sizes, a: Axial, b: Coronal CBCT images

Antero-posterior sizes of the lesions were measured on 0.3mm axial sections. Bucco-lingual and superior-inferior sizes of the lesions were measured on 1mm thickness coronal sections (Figure 3a and b).

#### Intensity

Of 10mm^2^ area in the middle of the CBCT coronal sectional image was evaluated according to new newtom software program Hounsfield unit (HU) scale measurement and the data were recorded separately for each lesion (Figure 3c).

### USG scanning and analyses of artificial mandible intraosseous lesions

#### USG Imaging Procedure

**Figure 4:**
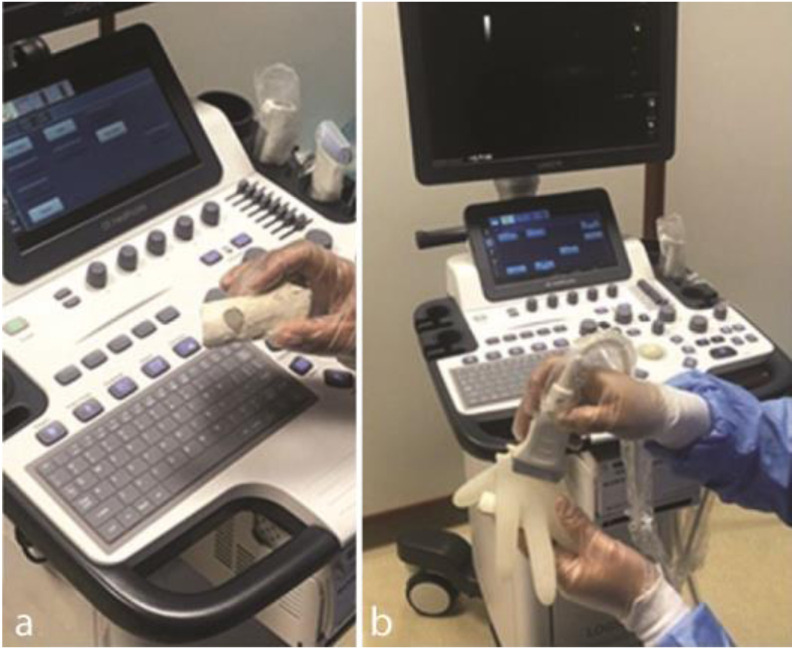
USG imaging procedure of the artificial mandible intraosseous lesion by placing the glove filled with water imitating soft tissue on the lesion

Artificial lesions in the study were evaluated with GE Logiq F8 (Jiangsu, China) USG device and 7-12 MHz linear array transducer probe. While scanning, medium size latex gloves filled with water were placed on the lesions, to smooth the scanned surface and to establish a good acoustic contact. Water based gel was applied on the surface of water filled glove surface corresponding to the lesion for a good acoustic contact and transversal and longitudinal images were taken (Figure 4).

### Evaluation of USG Images

**Figure 5:**
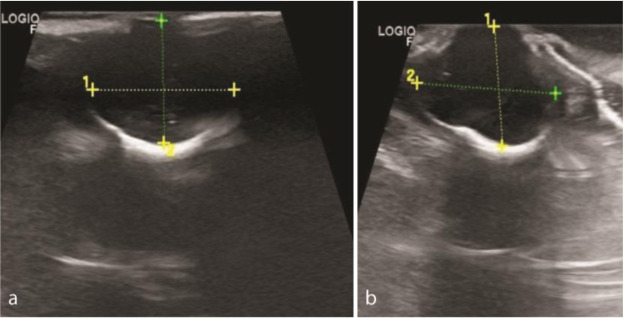
Size measurements of artificial lesion with USG, a: Number 1 measurement in anterior-posterior, probe transverse position, b: Number 1 bucco-lingual measurement and number 2 superior- inferior measurement, probe longitudinal

**Figure 6:**
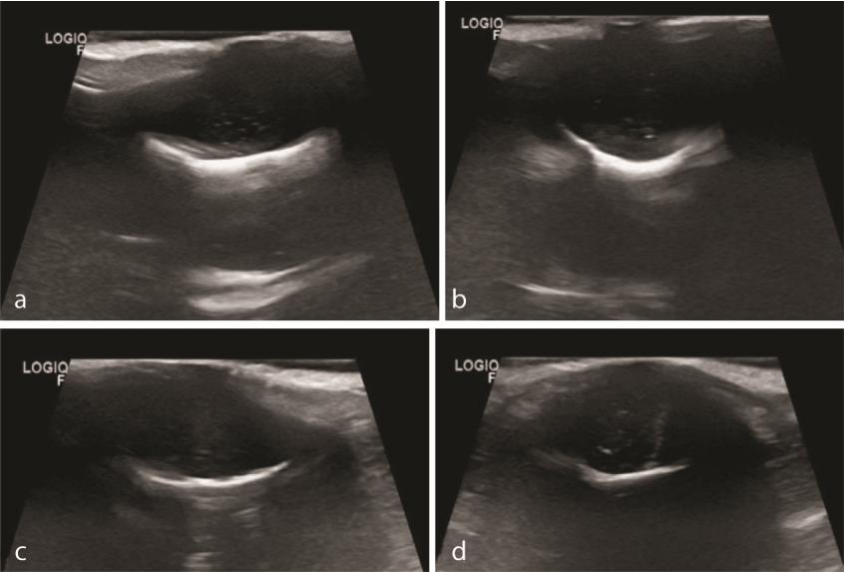
Lesion echogenicity a: Water, b: Milk, c: Olive oil anechoic, d: Liver hypoechoic appearance

In the USG images, the dimensions of the lesions were measured in anterior-posterior, bucco-lingual and superior-inferior sides (Figure 5) and the echogenicity of the lesions were recorded for each lesion (Figure 6). 

### Statistical Analysis

One sample t-test was used to statistically evaluate the difference between the averages size of the measurements. One-way Anova test was used to evaluate the difference between HU average values of the intensities of water, milk, olive oil, and liver groups, which were the materials that constituted the lesion content.

## Results

While the mean value of the anterior-posterior sizes of artificial lesions was found as 19.83±2.67mm in CBCT images, mean value of the same size was found as 18.7±3mm in USG images. Statistically significant difference was found between these two values (*p*=0.000) (Table 1). While the mean value of the bucco-lingual sizes of artificial lesions was found as 18.33±2.22mm in CBCT images, USG mean of the same size was found as 17.46 ±2.11mm. Statistically significant difference was found between these two values (*p*= 0.000) (Table 1).

**Table 1:** Comparison of artificial lesions dimensions with one Sample t- test

	Mean	Standart deviation	Mean difference	*P*
CBCT A-P	19.8383	2.67139	1.1300	0.000
USG A-P	18.7083	3.01815		
CBCT B-L	18.3300	2.22210	.86500	0.000
USG B-L	17.4650	2.11579		
CBCT SUP-INF	19.6950	2.17142	1.01500	0.000
USG SUP-INF	18.6800	2.38972		

While the mean value of the superior-inferior sizes of artificial lesions was found as 19.69±2.17mm in CB-CT images, mean value of the same size was found as 18.68±2.38mm in USG images. Statistically significant difference was found between these two values (*p*= 0.000) (Table 1).

The mean value of intensity of artificial lesions based on HU in CBCT images were found to be 174.6± 57.31 in water containing lesions, 175.8±51.22 in milk containing lesions, 174.13±88.81 in liver containing lesions, and -3.85±60.01 in oil containing lesions. There was a statistically significant difference between these values (*p*= 0.000) (Table 2). Moreover, the echogenicity of artificial lesions was anechoic for water, milk and olive oil, while it was hypoechoic for liver. 

**Table 2:** Comparison of artificial lesions contents HU values with CBCT

Lesion content	N	Mean	Standart Deviation	*P*
Milk	16	175.81^a^	51.223	0.000
Water	15	174.60^a^	57.318	
Liver	16	174.13^a^	88.819	
Olive oil	13	-3.85b	60.010	
Total	60	136.13	98.472	

One-way Anova, superscripts show statistically significant differen-ce between groups (Tukey testi, *p*< 0.0001) (a: no statistically significant, b: there is a statistically significant difference)

## Discussion

CBCT systems have been designed specifically for the maxillofacial region and in addition to being largely accessible for dentists, they have replaced CT in dental area due to their low radiation dose and cheaper installation and maintenance costs [ 18 - 20 ]. Besides these advantages, CBCT also has some disadvantages. CBCT radiation dose is higher when compared with two-dimensional imaging; soft tissue lesions cannot be evaluated correctly; HU correlation used as a standard in bone intensity assessment is limited, and artefacts can occur due to metal objects [ 21 - 22 ]. CBCT has valuable features such as providing the tools to examine the shape, region, borders and internal characteristics of intra-osseous lesions, trabecular feature of the surrounding bone and bucco-palatinal changes, and showing their relationship with neighbouring structures in detail [ 23 - 24 ]. When using USG, the image of structures behind the bone cannot be obtained normally. However, since many intraosseous lesions in jaws cause thinning or perforation, they allow the image be obtained with USG [ 25 ].

In addition to determining the content of the lesion, USG also allows getting information about the size of the lesion [ 26 - 27 ]. Musu *et al*. [ 28 ] evaluated the artificial bony lesions in bovine mandibular bone with USG and concluded that USG could be used regardless of the lesion diameter and buccal cortical condition (thickness, presence/absence of cortical plate). In this study, artificial lesions in plaster with cortical perforated were evaluated and lesion dimensions were compared between CBCT and USG. In addition, the HU unit was evaluated by CBCT and the echogenicity was assessed by USG.

Shahidi *et al*. [ 16 ] considered CBCT and CT images as gold standard for size measurement and they compared the largest size of lesions with USG. They found that CBCT and CT lesion sizes were larger than USG. Gundappa *et al*. [ 29 ] compared lesion sizes in USG with conventional and digital radiographies and they found lesion sizes to be smaller in almost all of the USG measurements. In their study, Bayrakdar *et al*. [ 30 ] compared intraosseous lesion sizes between the images obtained by CBCT and USG by measuring on three planes. While they did not find any statistical difference in mesiodistal and anterior-posterior measurements, they found difference in superior-inferior measurement, with USG measurements being smaller. In this study, measurements were made on three sides, statistical comparison was made, and statistical difference was found on three sides. In this study measurements comparisons were found to be in parallel with the superior-inferior measurements of Bayrakdar *et al*. [ 30 ], all measurements of Shahidi *et al*. [ 16 ] and Gundappa *et al*. [ 29 ]. The possible reasons of these differences might be due to the dissimilarities in cortex thickness over the lesion.

CT systems have a standard design that measures the attenuation of X-rays reaching body tissues called HU. HU can be used in the evaluation of bone in which the dental implants are inserted; it can be employed to control the grafts; and can provide beneficial information in diagnosis of lesions and anatomical structures [ 31 - 34 ]. Although CBCT systems give information about lesion content, it has been reported that they are not successful in differentiating between soft tissues with similar intensity since HU values used in the detection of intensity are not reliable [ 5 , 7 , 35 - 36 ]. While CBCT use of HU is not safe, there are soem studies performed on this field in literature [ 37 - 38 ]. Mah *et al*. [ 37 ] conducted a study by taking the images of a phantom containing eight different materials with 11 different CBCT and 2 different CT. In their study, they stated that HU scale could be used, which was obtained by calculating the attenuation coefficients obtained from the grey scale levels of dental CBCT scanners [ 37 ]. They reported that this situation would lead to positive developments in implant planning, surgical procedures, diagnosis, treatment planning, and in the reconstruction of two and three dimensional images in future for dentistry [ 37 ]. It has been reported that transforming grey scales of CBCT scanners into HU will have positive results for implant dentistry, cosmetic reconstruction, complex surgical treatment, and other dental treatments [ 37 ]. Buzatu *et al*. [ 38 ] orthodontically compared the HU values of midplatal suture before and after maxillary expansion treatment in CBCT system. In our study, HU values obtained from CBCT device were calculated according to different materials used. Average HU value was found 174.6 in artificial mandible intraosseous lesion containing water, 175.81 in those containing milk, 174.13 in those containing liver, and -3.85 in those containing olive oil. While statistically significant difference was found between oil and others, no significant difference was found between the other three materials (water, milk, liver). Our results brought to mind that HU values obtained from CBCT can differentiate oil in lesion content, while it cannot differentiate positive contents in HU scale and water.

 In USG method, internal echogenicity of the lesions give important information about the lesion. Cysts are seen in USG in anechoic, open-bound, and homogeneous echo features. However, if the cyst is infected, lesion content can be hypoechoic [ 39 - 40 ]. In their study, Shahidi *et al*. [ 16 ] reported that anechoic internal echogenicity was seen in radicular, residual, and dentigerous cystic lesions; hypoechoic internal echogenicity was seen in infected radicular cyst and odontogenic keratocyst. Moreover, they reported that hyperechoic internal echogenicity was seen in ameloblastoma, mural ameloblastoma and Pindborg tumour [ 16 ]. In our study, while artificial lesions containing water, milk and olive oil were found to have anechoic appearance, artificial lesions containing liver were found to have hypoechoic appearance. 

When compared with other medical methods, an important advantage of USG is finding out the vascularity of the lesion by using power Doppler and color Doppler feature, in addition to differentiating between cystic and solid in the lesion, it can differentiate between benign and malignant masses [ 41 ]. With USG, using power Doppler and color Doppler feature, granuloma and cyst can be distinguished in periapical lesions [ 42 ]. Since artificial mandible and artificial lesions were used in our study, lack of power Doppler imaging was considered as a limitation for this study. Other limitations were absence of soft tissue above the lesions and plaster and lesion contents were not vital materials.

## Conclusion

It was found that CBCT was superior to USG in dimensional evaluation of intraosseous lesions and it was seen again that CBCT is still the gold standard. Considering the differences in echogenicity, USG imaging could distinguish liquid form non-liquid contained lesions, while liquids with different intensity and features could not be differentiated from each other with echogenicity. It was thought that the HU system might be more sensitive especially in the negative values used in the CBCT system.
